# Performance of influenza case definitions for influenza community surveillance: based on the French influenza surveillance network GROG, 2009-2014

**DOI:** 10.2807/1560-7917.ES.2017.22.14.30504

**Published:** 2017-04-06

**Authors:** Jean-Sebastien Casalegno, Daniel Eibach, Martine Valette, Vincent Enouf, Isabelle Daviaud, Sylvie Behillil, Astrid Vabret, Jean Claude Soulary, Mehdi Benchaib, Jean Marie Cohen, Sylvie van der Werf, Anne Mosnier, Bruno Lina

**Affiliations:** 1 Univ Lyon, Virpath, CIRI, INSERM U1111, CNRS, ENS, Université Claude Bernard Lyon 1, Lyon, France; 2 These authors contributed equally; 3 National Reference Center for influenza viruses, National Influenza Center (southern France), Laboratory of Virology, Hospices Civils de Lyon, Lyon, France; 4 Bernhard Nocht Institute for Tropical Medicine (BNITM), Hamburg, Germany; 5 Coordinating Center of the National Reference Center for influenza viruses, National Influenza Center (northern France), Institut Pasteur, UMR 3569 CNRS, Université Paris Diderot Sorbonne Paris Cité, Paris, France; 6 Réseau des Groupes Régionaux d'Observation de la Grippe (GROG network); 7 OpenRome, Paris, France; 8 UNICAEN, EA4655 - U2RM, Normandie University, Caen, France; 9 Department of Virology, University Hospital, Caen, France; 10 Service de Médecine de la Reproduction, Hôpital Femme Mère Enfant, Hospices Civils de Lyon, Bron, France

**Keywords:** influenza, case definitions, surveillance, Europe, Influenza like illness

## Abstract

International case definitions recommended by the Centers for Disease Control and Prevention (CDC), the European Centre for Disease Prevention and Control (ECDC), and the World Health Organization (WHO) are commonly used for influenza surveillance. We evaluated clinical factors associated with the laboratory-confirmed diagnosis of influenza and the performance of these influenza case definitions by using a complete dataset of 14,994 patients with acute respiratory infection (ARI) from whom a specimen was collected between August 2009 and April 2014 by the Groupes Régionaux d’Observation de la Grippe (GROG), a French national influenza surveillance network. Cough and fever ≥ 39 °C most accurately predicted an influenza infection in all age groups. Several other symptoms were associated with an increased risk of influenza (headache, weakness, myalgia, coryza) or decreased risk (adenopathy, pharyngitis, shortness of breath, otitis/otalgia, bronchitis/ bronchiolitis), but not throughout all age groups. The WHO case definition for influenza-like illness (ILI) had the highest specificity with 21.4%, while the ECDC ILI case definition had the highest sensitivity with 96.1%. The diagnosis among children younger than 5 years remains challenging. The study compared the performance of clinical influenza definitions based on outpatient surveillance and will contribute to improving the comparability of data shared at international level.

## Introduction

According to the 2011 World Health Organization (WHO) guidelines, an influenza surveillance system aims to reliably detect the start and duration of the influenza season in order to monitor changes in the antigenicity of influenza viruses and provide guidance for influenza vaccine policies [1]. The system should provide continuous and robust data in order to monitor trends of clinically diagnosed influenza-like illness (ILI) and assess its disease burden in the general and high-risk population. The ability of the surveillance system to fulfil these epidemiological objectives depends on the accuracy of the clinical ILI case definition used. The search for the optimal case definition remains a public health challenge because of the lack of specificity of influenza symptoms, co-circulation of other respiratory viruses and low proportion of laboratory confirmation. Consequently, a variety of national case definitions are applied in surveillance networks worldwide, in addition to international ILI case definitions used by the United States (US) Centers for Disease Control and Prevention (CDC), the European Centre for Disease Prevention and Control (ECDC), and the WHO, which complicates data aggregation and comparison [2]. In addition to the established ILI case definitions, some surveillance systems use acute respiratory illness (ARI), a more sensitive but in exchange less specific case definition [2]. French influenza surveillance networks each have their own ILI definitions, which differ in the combination of clinical symptoms [2]. There are conflicting needs for a case definition: sensitive enough to ensure timely detection of the onset of an epidemic and specific enough to provide a small proportion of negative specimens among those tested and a robust impact estimate. The most accurate definition regarding sensitivity and specificity will provide the most accurate estimation of the number of influenza cases.

Evaluation and comparison of these case definitions are complicated by a variety of factors, such as differences in medical practice, prevalence during and outside the influenza seasons, respiratory co-infections in certain age groups, annual changes of influenza virus (sub)-types and heterogeneity of laboratory procedures for influenza testing. The optimal case definition should be applicable every year, internationally and in all medical settings (i.e community, outpatient and inpatient departments), regardless of the patients’ age or co-infections with co-circulating respiratory viruses such as respiratory syncytial virus (RSV) or rhinovirus [1]. Several previous studies have attempted to evaluate and compare the performance of the current ILI definitions, but are restricted either to a single hospital setting [3-8] or to cohort studies [9,10]. Only few studies have evaluated the performance of the current ILI/ARI definitions in the context of a national influenza sentinel network over several years [11,12] and none included a paediatric population. Based on the data collected between 2009 and 2014 by the Groupes Régionaux d’Observation de la Grippe (GROG), a French national influenza surveillance network, this study aimed to analyse clinical and non-clinical factors associated with the diagnosis of influenza and to compare the performance of international clinical case definitions.

## Methods

### GROG network

In France (population: 64.6 million), the surveillance of influenza is coordinated by the national public health agency, Santé publique France (formerly Institut de Veille Sanitaire (InVS)) and combines virological, clinical as well as community and hospital data [13]. The GROG was founded in 1984 according to WHO guidelines to detect the emergence of annual influenza virus outbreaks, to monitor changes in the antigenicity of influenza viruses, to guide the selection of strains for the annual influenza vaccine, and to provide virus samples for use in vaccine production [14]. This network comprises 548 volunteer practitioners, 112 paediatricians and nine laboratories (two reference laboratories and seven hospital virology laboratories) distributed in all 22 regions of metropolitan France.

The sentinel physicians participating in the GROG network reported the weekly number of patients with acute respiratory infection (ARI), as defined by the GROG, presenting at their practice during the active influenza surveillance period (week 40 to 15). They collected information and provided, on a random sampling basis, nasal/pharyngeal swabs from a subset of ARI patients presenting within 48 hour of symptom onset. The definition of ARI adopted by the GROG was as follows: sudden onset of at least one respiratory sign (e.g. cough, sore throat, shortness of breath, coryza) AND at least one general symptom suggestive of an acute infectious disease (e.g. fever, fatigue, headache, malaise) (Table 1).

**Table 1:** Case definitions, three international ILI definitions and one national ARI definition (used as inclusion criterion), GROG study, France, 2009–2014

Definition	Type	Sudden onset	General symptoms	Respiratory symptoms
ECDC	ILI	Yes	At least one among: fever, feverishness, headache, malaise, myalgia	At least one among: cough, sore throat^a^, shortness of breath
WHO	ILI	No	Fever ≥ 38 °C with onset within the last 10 days	Cough
CDC	ILI	Yes	Fever ≥ 100° F (37.8 °C)^b^ Absence of a known cause other than influenza	At least one among: cough, sore throat^a^
GROG	ARI	Yes	At least one among: fever ≥ 38 °C, headache, weakness, myalgia, chills	At least one among: cough, coryza, bronchitis, pharyngitis, shortness of breath, expectoration

ARI: Acute respiratory illness; CDC: Centers for Disease Control and Prevention; ECDC: European Centre for Disease Prevention and Control; GROG: Groupes Régionaux d’Observation de la Grippe; ILI: influenza-like illness; WHO: World Health Organization.

^a^ The sore throat symptom is not collected in the GROG network. For the purpose of this work, the variable was replaced by pharyngitis diagnosis.

^b^ Fever is defined in the GROG network as a temperature fever ≥ 100.4°F (38.0 °C). For the purpose of this work, fever ≥ 100° F (37.8 °C) was replaced by fever ≥ 100.4°F (38.0 °C).

Fever was defined as a body temperature greater than or equal to 38 °C. For each patient sampled, a standardised case reporting form was completed and sent along with the specimen to the corresponding reference or hospital laboratory. Influenza A and B viruses were detected by real-time RT-PCR [15]. The influenza A virus subtype (H1N1pdm09 or H3N2) was further determined by RT-PCR as provided by the Coordinating Centre of the National Reference Centre for influenza viruses (data not shown). All participating laboratories validated their assays appropriately.

### Study database

All cases between 2009 and 2014 were extracted from the GROG database. Patients were excluded from the study database if their specimens were positive for two influenza virus (sub)types or for influenza C virus, if they were sampled more than 48 hours after the onset of symptoms, or if at least one variable required for the analysis was incomplete. To avoid any inclusion bias in the patient selection, patients were excluded if the symptoms did not meet the GROG ARI definition. The start and the end of the influenza pandemic, the seasonal influenza epidemics and the bronchiolitis epidemics were defined by Santé publique France (former InVS) on the basis of the national surveillance network. A confirmed case of influenza was defined as a patient with a positive laboratory result for influenza A or B viruses.

### Database analysis

All patients included in the study database were described by sex and age. Continuous variables were summarised as means with standard deviation (median with interquartile range (IQR) for non-normally distributed variables), and dichotomous or categorical variables were summarised as percentages. Influenza positivity rates were calculated by age group and month of the year.

A generalised estimating equation model was used to take account of the potential clustering of observations by practitioners. We fit a one-level, hierarchical, logistic regression model that incorporated the practitioner identity variables (level 1) using the SPSS V19 (IBM, Chicago, US) GENLIN function. Firstly, univariate associations describing the relationship of each potential predictive factor (sex, temperature, clinical symptoms, clinical case definition) with the outcome of laboratory-confirmed influenza, were examined with univariate logistic regression analysis. Secondly, multivariable logistic regression models were used to investigate the combined influence of clinical variables tested in the bivariate analysis (sex, temperature, clinical symptoms) as potential independent predictive factors for laboratory-confirmed Influenza. In the non-stratified multivariate analysis, the interaction terms concerning the age group were also introduced in the models to adjust for the potential bias.

Both univariate and multivariate analyses were stratified according to age group. In the stratified and non-stratified multivariate analysis, influenza epidemic, influenza pandemic and bronchiolitis period were introduced as variables to adjust for potential bias.

Sensitivity, specificity and area under the curve (AUC) were calculated to assess the performance of case definitions by age group (0–4, 5–14, 15–64, ≥ 65 years) and influenza (sub)type (influenza A(H1N1)pdm09, A(H3N2) and influenza B). Sensitivity was defined as the proportion of laboratory-confirmed influenza patients who fulfilled the clinical case definition. The specificity was defined as the proportion of influenza-negative patients who did not fulfil the clinical case definition. The average predictive performance was quantified using the area under the receiver operating characteristic curve to determine the AUC.

A p value below 0.05 was considered significant. The statistical analysis for the GROG database was performed with SPSS v19 (IBM, Chicago, US) software.

### Case definitions tested

We selected the three most commonly used international ILI definitions [1,2]: the ECDC ILI definitions, the WHO ILI definition updated in 2011 and the CDC ILI definition (Table 1). All definitions include the presence of general (e.g. fever) and respiratory symptoms with or without a sudden onset. The number of included criteria varies from three (WHO) to nine (ECDC).

### Ethics

Oral informed consent was obtained from patients at the moment of swab taking in accordance with national regulations. All swab results and forms were anonymised by the laboratories before they were sent to the GROG network coordination. In accordance with applicable laws and regulations, no clearance by an Ethics Committee is required in France for the retrospective analysis of anonymised data collected within routine influenza surveillance schemes.

## Results

### Database description

The work was conducted on a complete dataset of 14,994 patient specimens collected between August 2009 and April 2014. This includes the 2009 influenza pandemic and the four seasonal influenza epidemics 2010/11 to 2013/14. Of those patients, 38.7% (5,806/14,994) tested positive for influenza, 29.1% (4,370/14,994) for influenza A and 9.6% (1,436/14,994) for influenza B. For influenza A cases, A(H1N1)pdm09 viruses and A(H3N2) viruses were detected in 18.9% (2,837/14,994) and 10.2% (1,533/ 14,994) respectively.

Influenza A(H1N1)pdm09 viruses predominated during the 2009/10, 2010/11 and 2013/14 influenza seasons, while influenza B viruses predominated in the 2010/11 and the 2012/13 season and A(H3N2) viruses predominated during the 2011/12 season.

The database consisted of 51.2% (7,674/14,994) male patients. The median age of all cases was 9 years (IQR: 3–31 years), increasing to 10 (IQR: 4–31 years) among influenza-positive patients. The patients mostly belonged to the paediatric population, with 60.7% (9,103/14,994) being younger than 15 years (Table 2).

**Table 2:** Influenza-positive and negative patients included in the study, by male sex, age distribution, temperature group, with clinical symptoms and by period, GROG study, France, 2009­–2014 (n = 14,994)

	All cases n = 14,994		Patients with laboratory-confirmed influenza n = 5,806		Patients who tested negative for influenza n = 9,188	
	n	%	n	%	n	%
**Sex**						
Male sex: n (%)	7,674	51.2	3,004	51.7	4,670	50.8
**Age distribution (years)**						
0–4	5,521	36.8	163	28.2	3,886	42.3
5–14	3,582	23.9	1,897	32.7	1,685	18.3
15–64	5,338	35.6	2,074	35.7	3,264	35.5
≥ 65	553	3.7	200	3.4	353	3.8
Mean age (standard deviation)	18.5 (± 20.6)		19.1 (± 20.0)		18.2 (± 21.0)	
Median age (interquartile range)	9.0 (3.0–31.0)		10.0 (4.0–31.0)		7.0 (2.0–32.0)	
**Temperature group (**°C**)**						
T < 38	636	4.2	155	2.7	481	5.2
38 ≤ T < 38.5	1,868	12.5	553	9.5	1,315	14.3
38.5 ≤ T < 39	5,159	34.4	2,000	34.5	3,159	34.4
T ≥ 39	7,331	48.9	3,098	53.3	4,233	46.1
**Clinical symptoms**						
Cough	12,476	83.2	5,224	90.0	7,252	78.9
Headache	8,389	55.9	3,683	63.4	4,706	51.2
Weakness	11,424	76.2	4,754	81.9	6,670	72.6
Chills	8,735	58.3	3,766	64.9	4,969	54.1
Myalgia	8,512	56.8	3,643	62.8	4,869	53.0
Coryza/rhinitis	11,064	73.8	4,360	75.1	6,704	73.0
Conjunctivitis	1,320	8.8	522	9.0	798	8.7
Gastrointestinal symptoms	2,949	19.7	1,166	20.1	1,783	19.4
Adenopathy	1,625	10.8	596	10.3	1,029	11.2
Pharyngitis	8,424	56.2	3,109	53.6	5,315	57.9
Shortness of breath	1,319	8.8	428	7.4	891	9.7
Otitis/otalgia	1,544	10.3	469	8.1	1,075	11.7
Bronchitis	1,324	8.8	399	6.9	925	10.1
Rash	98	0.7	22	0.4	76	0.8
**Period**						
RSV bronchiolitis period	6,444	43.0	2,228	38.4	4,216	45.9
Pandemic period	4,282	28.6	1,534	26.4	2,748	29.9
Seasonal period	6,470	43.2	2,939	50.6	3,531	38.4

GROG: Groupes Régionaux d’Observation de la Grippe; RSV: respiratory syncytial virus; T: temperature.

In all age groups, A(H1N1)pdm09 was the most prevalent influenza virus (respective prevalence value for the 0–4, 5–14 and 15–64 years age group: 13.5% (747/5,521), 27.3% (978/3,582), 20.1% (1,075/5,338)) except for the ≥ 65 years age group with a prevalence of 6.7% (37/553). Influenza A(H3N2) was the most prevalent influenza virus in the ≥ 65 years age group (prevalence: 21.2% (117/553)) and the second most prevalent in the 15–64 and 0–4 years age groups (respective prevalence: 10.6% (568/5,338) and 9.5% (524/5,521)). Influenza B was the second most prevalent influenza virus in the 5–14 years age group (prevalence: 16.6% (595/3,582)).

### Clinical and demographic predictors of laboratory-confirmed influenza detection

The most predictive clinical symptoms for laboratory-confirmed influenza were cough (odds ratio (OR) = 2.40; 95% confidence interval (CI): 2.11–2.72), temperature ≥ 39 °C (OR = 2.27; 95% CI: 1.85–2.79) or between 38.5 °C and 39 °C (OR = 1.96; 95% CI: 1.61–2.40), and weakness (OR = 1.71; 95% CI: 1.54–1.90) (Table 3).

**Table 3:** Clinical signs and symptoms associated with laboratory-confirmed influenza, stratified by age group, GROG study, France, 2009–2014 (n = 14,994)

Variable	0–4 years (n = 5,521)			5–14 years (n = 3,582)			15–64 years (n = 5,338)			≥ 65 years (n = 553)			Total (n = 14,994)		
	OR	95% CI	P value	OR	95% CI	p value	OR	95% CI	P value	OR	95% CI	P value	OR	95% CI	p value
**Sex**															
Male	Reference			Reference			Reference			Reference			Reference		
Female	NS			NS			NS			NS			NS		
**Temperature group (**°C**)**															
T < 38	Reference			Reference			Reference			Reference			Reference		
38 ≤ T < 38.5	NS			NS			1.37	1.05–1.81	0.023	NS			1.30	1.05–1.62	0.015
38.5 ≤ T < 39	2.78	1.33–5.81	0.006	1.55	1.02–2.34	0.039	2.20	1.68–2.89	<0.001	2.08	1.11–3.92	0.023	1.96	1.61–2.40	<0.001
T ≥ 39	3.55	1.74–7.22	<0.001	2.05	1.34–3.16	0.001	2.69	2.07–3.50	<0.001	3.18	1.54–6.58	0.002	2.27	1.85–2.79	<0.001
**Clinical parameters**															
Cough	1.41	1.17–1.69	<0.001	3.27	2.71–3.96	<0.001	3.72	3.01–4.60	<0.001	3.93	1.92–8.05	<0.001	2.40	2.11–2.72	<0.001
Headache	1.65	1.43–1.92	<0.001	1.23	1.06–1.43	<0.001	NS			NS			1.65	1.51–1.81	<0.001
Weakness	1.59	1.36–1.85	<0.001	1.55	1.33–1.80	<0.001	1.36	1.09–1.68	0.006	1.64	1.05–2.57	0.030	1.71	1.54–1.90	<0.001
Chills	1.38	1.21–1.60	<0.001	1.33	1.15–1.54	<0.001	1.76	1.52–2.04	<0.001	NS			1.57	1.44–1.71	<0.001
Myalgia	1.55	1.32–1.82	<0.001	1.26	1.10–1.44	0.001	1.26	1.03–1.55	0.025	NS			1.49	1.36–1.64	<0.001
Coryza/rhinitis	NS			1.39	1.19–1.62	0.001	1.24	1.07–1.45	0.005	NS			1.12	1.02–1.23	0.022
Conjunctivitis	NS			NS			1.27	1.02–1.59	0.032	NS			NS		
Gastrointestinal symptoms	1.19	1.03–1.38	0.018	NS			NS			NS			NS		
Adenopathy	NS			NS			0.74	0.59–0.93	0.010	NS			NS		
Pharyngitis	0.81	0.70–0.94	0.006	0.81	0.70–0.94	0.005	0.85	0.75–0.96	0.010	NS			0.84	0.77–0.91	<0.001
Shortness of breath	0.43	0.31–0.61	<0.001	NS			NS			NS			0.74	0.64–0.86	<0.001
Otitis/otalgia	0.68	0.57–0.81	<0.001	0.70	0.53–0.92	0.010	NS			NS			0.66	0.59–0.75	<0.001
Bronchitis	0.36	0.28–0.47	<0.001	NS			1.33	1.03–1.71	0.028	NS			0.66	0.54–0.81	<0.001
Rash	0.34	0.17–0.71	0.004	NS			NS			NS			0.46	0.29–0.71	0.001

CI: confidence interval; GROG: Groupes Régionaux d’Observation de la Grippe; NS: statistically not significant; OR: odds ratio; T: temperature.

Univariate analysis was performed using a generalised estimating equation model to account for the potential clustering of observations by general practitioner. Only cough, weakness and a temperature > 38.5 °C were significantly associated with influenza across all age groups. Notably, the symptom cough revealed increasing ORs with increasing age (0–4 years: OR = 1.41; ≥ 65 years: OR = 3.93) and bronchitis was associated with influenza in the 15–64 years age group (OR = 1.33; 95% CI: 1.03–1.71), while it was negatively associated in the 0–4 years age group (OR = 0.36; 95% CI: 0.28–0.47).

All factors were entered into the multiple regression model performed on the whole database and stratified by age groups (Table 4). Multivariate analysis was performed using a generalised estimating equation model to account for the potential clustering of observations by general practitioner. All the variables tested in the univariate analysis were included in the multivariate analysis. Only results from the variables that were significant (p < 0.05) in the multivariate analysis are shown in the table.

**Table 4:** Clinical signs and symptoms associated in multivariate analysis with laboratory-confirmed influenza, stratified by age group, GROG study, France, 2009–2014 (n = 14,994)

Variable	0–4 years (n = 5,521)			5–14 years (n = 3,582)			15–64 years (n = 5,338)			≥ 65 years (n = 553)			Total (n = 14,994)		
	OR	95% CI	p value	OR	95% CI	p value	OR	95% CI	p value	OR	95% CI	p value	OR	95% CI	p value
**Sex**															
Male	Reference			Reference			Reference			Reference			Reference		
Female	NS			NS			NS			NS			NS		
**Temperature group (**°C**)**															
T < 38	Reference			Reference			Reference			Reference			Reference		
38 ≤ T < 38.5	NS			NS			1.34	1.01–1.76	0.040	2.47	1.05–5.82	0.039	1.32	1.06–1.65	0.014
38.5 ≤ T < 39	NS			NS			2.06	1.55–2.74	<0.001	2.33	1.17–4.68	0.017	1.95	1.59–2.41	<0.001
T ≥ 39	2.51	1.26–5.0	0.009	2.08	1.32–3.30	0.002	2.57	1.95–3.40	<0.001	3.61	1.66–7.89	0.001	2.50	2.02–3.10	<0.001
**Clinical symptoms **															
Cough	1.62	1.35–1.95	<0.001	3.06	2.50–3.78	<0.001	3.63	2.92–4.51	<0.001	5.55	2.67–11.52	<0.001	2.53	2.23–2.90	<0.001
Headache	1.37	1.19–1.57	<0.001	NS			NS			NS			1.20	1.10–1.31	<0.001
Weakness	1.39	1.20–1.61	<0.001	1.50	1.25–1.80	<0.001	NS			1.85	1.11–3.07	0.018	1.37	1.24–1.53	<0.001
Chills	NS			NS			1.51	1.28–1.79	<0.001	0.62	0.40–0.96	0.030	NS		
Myalgia	1.20	1.03–1.40	0.020	NS			NS			NS			1.18	1.06–1.30	0.002
Coryza/rhinitis	NS			1.39	1.17–1.65	<0.001	1.32	1.13–1.55	0.001	NS			1.26	1.14–1.39	<0.001
Conjunctivitis	NS			NS			NS			NS			NS		
Gastrointestinal symptoms	NS			0.83	0.69–1.00	0.047	0.83	0.71–0.98	0.024	NS			NS		
Adenopathy	NS			NS			0.72	0.56–0.92	0.009	NS			0.82	0.71–0.94	0.004
Pharyngitis	0.81	0.71–0.93	0.003	0.81	0.68–0.96	0.013	NS			NS			0.86	0.79–0.93	<0.001
Shortness of breath	0.58	0.42–0.78	<0.001	0.69	0.49–0.96	0.024	NS			NS			0.54	0.40–0.73	<0.001
Otitis/otalgia	0.71	0.59–0.85	<0.001	NS			NS			NS			0.75	0.66–0.85	<0.001
Bronchitis	0.45	0.35–0.59	<0.001	NS			NS			NS			0.43	0.34–0.54	<0.001
Rash	0.38	0.18–0.82	0.014	NS			NS			NS			NS		

CI: confidence interval; GROG: Groupes Régionaux d’Observation de la Grippe; NS: statistically not significant; OR: odds ratio; T: temperature.

In the non-stratified and stratified multivariate analyses, influenza epidemic, influenza pandemic and bronchiolitis period were introduced as variables to adjust for potential bias. In the non-stratified multivariate analysis, the interaction terms concerning the age group were also introduced in the models to adjust for the potential bias.

Temperature was independently associated with influenza. ORs increased with rising body temperature, from 1.32 (95% CI: 1.06–1.65; 38–38.5 °C) to 2.50 (95% CI: 2.02–3.10; ≥ 39 °C). Only a body temperature ≥ 39 °C and cough were associated with laboratory-confirmed influenza across all age groups. Associations with cough seemed to increase with age, being more predictive among the ≥ 65 year-olds (OR = 5.55; 95% CI: 2.67–11.52) and weaker among 0–4 year-olds (OR = 1.62; 95% CI: 1.35–1.95). The clinical symptoms chills, conjunctivitis and gastrointestinal symptoms were not predictive and dropped out of the final multivariate model.

The multivariate model varied tremendously by age group. In the 0–4 years age group, four symptoms were positively associated (OR range: 1.20–1.62) and five were negatively associated (OR range: 0.38–0.81) with influenza infection. In the ≥ 65 years group, only cough (OR = 5.55; 95% CI: 2.67–11.52) and weakness (OR = 1.85; 95% CI; 1.11–3.07) were positively associated, and chills (OR = 0.62; 95% CI: 0.40–0.96) were negatively associated with influenza infection.

### Performance of current ILI and ARI definitions

When testing the performance of the case definitions, the WHO ILI case definition revealed by far the highest specificity with 21.4%, while the ECDC ILI and CDC ILI case definitions had the highest sensitivity with, respectively, 96.1% and 95.7% (Table 5).

**Table 5:** Sensitivity, specificity and area under curve value of the case definitions tested for detection of influenza, GROG study, France, 2009–2014

Case definition	Sensitivity % (95% CI)	Specificity % (95% CI)	AUC % (95% CI)
ECDC ILI	96.1 (95.5–96.6)	6.6 (6.1–7.1)	0.513 (0.504–0.523)
CDC ILI	95.7 (95.2–96.2)	7.3 (6.8–7.9)	0.515 (0.506–0.524)
WHO	89.8 (89.0–90.6)	21.4 (20.6–22.3)	0.556 (0.547–0.566)

AUC: area under curve; CDC: Centers for Disease Control and Prevention; CI: confidence interval; ECDC: European Centre for Disease Prevention and Control; GROG: Groupes Régionaux d’Observation de la Grippe; ILI: influenza-like illness; WHO: World Health Organization.

The WHO case definition was the most discriminant definition with the highest positive AUC values (AUC = 0.556; 95% CI: 0.547–0.566) compared with the ECDC ILI (AUC = 0.513; 95% CI; 0.504–0.523) and CDC ILI (AUC = 0.515; 95% CI: 0.506–0.524) definition.

### Impact of age group, influenza (sub)type and epidemic period on performance of current ILI and ARI definitions

All ILI case definitions presented with the lowest sensitivity among the 0–4 years age group (Table 6) and the highest sensitivity among the ≥ 65 years age group.

**Table 6:** Sensitivity, specificity, and area under curve value of the case definitions tested for detection of influenza, stratified by age group and influenza (sub)-type, GROG study, France, 2009–2014

	Stratification variable	ECDC ILI	WHO	CDC
Sensitivity % (95% CI)				
Age group	0–4 years	93.4 (92.1–94.5)	84.2 (82.3–85.9)	93.3 (91.9–94.4)
	05–14 years	95.8 (94.8–96.7)	89.8 (88.4–91.1)	95.4 (94.4–96.3)
	15–64 years	98.2 (97.5–98.7)	93.7 (92.6–94.7)	97.7 (97.0–98.3)
	≥ 65 years	98.5 (95.7–99.7)	96.0 (92.3–98.3)	98.0 (95.0–99.5)
Influenza type	A(H1N1) pdm09	96.9 (96.2–97.5)	91.8 (90.7–92.8)	96.7 (95.9–97.3)
	A(H3N2)	95.6 (94.4–96.5)	89.2 (87.6–90.7)	95.0 (93.8–96.1)
	B	94.9 (93.6–96.0)	86.6 (84.7–88.3)	94.6 (93.2–95.7)
Epidemic Period	RSV bronchiolitis	96.7 (95.8–97.4)	90.1 (88.8–91.3)	96.1 (95.2–96.9)
	Influenza pandemic^a^	97.5 (96.5–98.2)	92.5 (91.0–93.8)	97.2 (96.2–97.9)
	Influenza seasonal^b^	95.5 (94.8–96.2)	88.9 (87.8–89.9)	95.2 (94.4–95.9)
Specificity % (95% CI)				
Age group	0–4 years	7.7 (6.9–8.7)	21.0 (19.7–22.3)	8.0 (7.2–8.9)
	05–14 years	8.5 (7.2–10.0)	27.1 (25.0–29.3)	9.0 (7.7–10.5)
	15–64 years	4.3 (3.7–5.1)	19.9 (18.5–21.3)	5.7 (7.7–10.5)
	≥ 65 years	4.8 (2.9–7.8)	13.9 (10.5–18.0)	6.5 (4.3–9.8)
Influenza type	A(H1N1) pdm09	6.6 (6.1–7.1)	21.4 (20.6–22.3)	7.3 (6.8–7.9)
	A(H3N2)	6.6 (6.1–7.1)	21.4 (20.6–22.3)	7.3 (6.8–7.9)
	B	6.6 (6.1–7.1)	21.4 (20.6–22.3)	7.3 (6.8–7.9)
Epidemic period	RSV bronchiolitis	6.6 (5.9–7.5)	19.3 (18.1–20.5)	7.6 (6.8–8.4)
	Influenza pandemic^a^	6.1 (5.2–7.1)	18.2 (16.8–19.7)	7.0 (6.1–8.1)
	Influenza seasonal^b^	7.0 (6.2–8.0)	21.3 (19.8–22.8)	7.8 (6.8–8.8)
AUC % (95% CI)				
Age group	0–4 years	0.506 (0.489–0.522)	0.526 (0.509–0.542)	0.507 (0.490–0.523)
	05–14 years	0.522 (0.503–0.541)	0.585 (0.566–0.604)	0.522 (0.503–0.541)
	15–64 years	0.512 (0.497–0.528)	0.568 (0.553–0.583)	0.517 (0.501–0.533)
	≥ 65 years	0.517 (0.467–0.566)	0.549 (0.501–0.598)	0.523 (0.473–0.572)
Influenza type	A(H1N1) pdm09	0.517 (0.505–0.529)	0.566 (0.555–0.578)	0.520 (0.508–0.532)
	A(H3N2)	0.517 (0.495–0.526)	0.553 (0.539–0.568)	0.512 (0.496–0.527)
	B	0.507 (0.491–0.523)	0.540 (0.525–0.555)	0.509 (0.493–0.525)
Epidemic period	RSV bronchiolitis	0.517 (0.502–0.531)	0.547 (0.532–0.561)	0.518 (0.504–0.533)
	Influenza pandemic^a^	0.518 (0.500–0.536)	0.553 (0.536–0.571)	0.521 (0.503–0.539)
	Influenza seasonal^b^	0.513 (0.499–0.527)	0.551 (0.537–0.565)	0.515 (0.501–0.529)

AUC: area under curve; CDC: Centers for Disease Control and Prevention; CI: confidence interval; ECDC: European Centre for Disease Prevention and Control; GROG: Groupes Régionaux d’Observation de la Grippe; ILI: influenza-like illness; RSV: respiratory syncytial virus; WHO: World Health Organization.

^a^ Influenza A(H1N1)pdm09 sample during the 2009 influenza pandemic.

^b^ Influenza A(H1N1)pdm09 and A(H3N2) sample during seasonal influenza epidemics (2010/11, 2011/12, 2012/13, 2013/14).

The WHO definition revealed the largest sensitivity difference (11.8%) between the oldest and the youngest age groups and had the poorest sensitivity in the 0–4 years age group (84.2%). There was no noticeable difference in sensitivities between the three definitions in the ≥ 65 years age group. Stratified by influenza (sub)type, the ECDC and CDC definitions performed similarly with sensitivities above 94%, while the WHO ILI had a higher sensitivity for influenza A(H1N1)pdm09 (91.8%) and A(H3N2) (89.2%) than for influenza B (86.6%). Stratified by influenza period, all ILI case definitions showed highest sensitivities during the pandemic period compared with the epidemic periods.

Accordingly, all ILI case definitions showed the highest specificity among the 5–14 year-olds, and the WHO definition had the highest specificity in all age groups. Stratified by influenza period, the ECDC and CDC definitions had similar specificity, while the WHO ILI had a higher specificity during the influenza seasonal epidemic period compared with the pandemic periods.

All definitions revealed the highest AUC values among the 5–14 year-olds and for the A(H1N1)pdm 09 viruses. The WHO definition had the highest AUC values in all age groups, all influenza (sub)types and all tested periods (influenza pandemic, influenza epidemics and bronchiolitis period). There was no significant difference in the AUC values for each definition among the three tested periods.

## Discussion

This study evaluated both the clinical factors associated with the diagnosis of influenza and the performance of influenza case definitions, based on a national influenza surveillance database. The database had distinct features: (i) influenza was confirmed by the gold standard RT-PCR technique, (ii) the database included a large paediatric population and (iii) the database covered one pandemic and four seasonal influenza epidemics, with information on influenza A and B viruses.

This study identified cough and fever ≥ 39 °C as the symptoms which most accurately predicted an influenza infection in all age groups. Similar findings have been reported previously [9,12]. Several other symptoms (headache, weakness, myalgia, coryza) were associated with an increased risk of influenza infection but not throughout all age groups. On the other hand, pharyngitis appeared to be associated with a decreased risk of influenza infection in all age group except those 65 years and older. Assuming an overlap between the variables pharyngitis and sore throat, these two symptoms might not improve, but rather weaken an ILI definition. This result supports the updated WHO definition from 2011 that removed sore throat from its definition [1]. Based on this study and the current literature, we believe that there is evidence to exclude ‘sore throat’ from ILI definitions (such as ECDC and CDC ILI definition). Several others symptoms (adenopathy, pharyngitis, shortness of breath, otitis/otalgia, bronchitis/bronchiolitis, rash) were associated with a decreased risk of influenza infection, but not in all age groups. Surprisingly, shortness of breath also appeared to be associated with a decreased risk of influenza in the younger patients (younger than 14 years). This result suggests that this symptom may contribute to weaken the performance of the ECDC and CDC ILI definitions in the younger age groups and may also rather be excluded from ILI definitions.

Negative associations at age 0–4 years could be due to other respiratory tract pathogens circulating in this age group [16]. The variety of other potential co-infecting pathogens may have caused the lower performance of all case definitions in the 0–4 years age group [10]. One way to improve the specificity of the ILI definition in this particular age group would be a higher temperature cut-off because the multivariate model showed that in the youngest age group, only high body temperatures above 38.5 °C were strongly associated with influenza.

These strong age-dependent differences are likely to have contributed substantially to the variable performance of case definitions reported in different studies, in particular when the age groups 0–4 years and ≥ 65 years are underrepresented in the tested population [17]. In addition, it remains difficult to measure the impact of influenza types or subtypes as they are tightly associated with the age group. For example, stratification by Influenza virus (sub)types showed that the WHO ILI definitions had lower sensitivity for influenza B. Indeed, to our knowledge, no differences in clinical symptoms have been reported so far for outpatients infected with influenza A compared with influenza B viruses [18]. Hence, age is probably the main confounding factor as most of the patients with influenza B in our study were 5–14 years-old, whereas patients with influenza A were predominantly 0–4 years-old. Therefore the evolving epidemiology of influenza may indirectly impact the performance of surveillance networks. Those results strongly suggest that interpretation of syndromic surveillance data without information on age may be misleading [17]. It is very unlikely that a ‘one size fits all’ approach reaches optimal performance for all the age groups or influenza (sub)type. To do so, it may be necessary to develop age or (sub)type-specific case definitions for influenza. The temperature cut-off may be adjusted, notably in the older and younger age groups, as it greatly impacted the sensitivity and specificity [19].

Our study had some limitations. It should be noted that the case definitions were tested with those variables which were collected by the surveillance network. In the present study, the clinical diagnosis pharyngitis was used instead of the case definition variable sore throat, which might have resulted in some discrepancies, and interpretation must be done cautiously. Fever was defined as a body temperature ≥ 38 °C for all case definitions, although the ECDC ILI definition does not define any exact temperature cut-off and the CDC ILI definition defines fever for a temperature ≥ 100° F (37.8 °C). This slight alteration of the CDC definition should be taken into account when interpreting the study results. However, the impact of such an alteration should be minimal compared with other known factors that affect the measurement of body temperature such as: individual variability, daily variation, site of measurement and the natural trend for physicians to round up or round down temperatures to .5 or .0 digits (i.e in the case of American doctors the 100 °F and European doctors 38 °C [20].

Due to the predefined temperature cut-off of the GROG database, sub- or afebrile patients in our database who did not also present headache, weakness, myalgia or chills were not included. Therefore we cannot exclude that sensitivity may have been over- and specificity underestimated. These results are in accordance with data obtained by Thurksy et al. in a similar setting (the Australian influenza surveillance programme) and in the absence of a defined temperature cut-off for fever [21]. Indeed Thurksy et al. reported, over two influenza seasons, a high sensitivity (98.4–100.0%) and a very low specificity (7.1–12.9%) for the CDC definition. However, the performance results in our study differed from other studies, which relied either on hospitalised patients [7,8] or on a cohort of self-reporting adults [22] that observed higher specificity and lower sensitivity values for similar clinical definitions.

It is still questionable to what extent the results could be applied to other surveillance systems. Indeed, the patients were sampled according to the GROG case definition, which may have influenced the results. In general, it is challenging to fully investigate the relation between clinical features and healthcare seeking behaviour that strongly determine the characteristics of the study population (demographic and clinical) and most probably impact the performance of a case definition, as already suggested by Jiang et al. [22]. Another open question is how these surveillance definitions will perform in the context of an influenza epidemic caused by an emerging influenza virus with more atypical clinical symptoms, for example conjunctivitis in the context of infection with an avian influenza virus.

## Conclusions

The study compares the performance of clinical influenza definitions in the setting of a national network based on outpatient surveillance. The revised WHO ILI definition could be chosen for surveillance purposes for its higher specificity and better performance in all age groups, which allowed a more accurate estimation of influenza case numbers and an increase in the proportion of influenza-positive samples. In any case, the diagnosis among children younger than 5 years remains challenging, as only fever was highly predictive of influenza infection, suggesting that the temperature cut-off in the case definition is critical to accurately predict influenza among the large number of differential diagnoses in that age group.
